# Modeling of Hepatocytes Proliferation Isolated from Proximal and Distal Zones from Human Hepatocellular Carcinoma Lesion

**DOI:** 10.1371/journal.pone.0153613

**Published:** 2016-04-13

**Authors:** Mauro Montalbano, Giuseppe Curcurù, Ali Shirafkan, Renza Vento, Cristiana Rastellini, Luca Cicalese

**Affiliations:** 1 Department of Surgery, University of Texas Medical Branch, Galveston, Texas, United States of America; 2 Department of Experimental Biomedicine and Clinical Neurosciences, University of Palermo, Via del Vespro 129, Polyclinic, 90127 Palermo, Italy; 3 Department of Chemical, Management, Informatics and Mechanical Engineering, University of Palermo, Palermo, Italy; 4 Laboratory of Biochemistry, Department of Biological, Chemical and Pharmaceutical Sciences and Technologies, Polyclinic, University of Palermo, Palermo, Italy; 5 Institute for Cancer Research and Molecular Medicine and Center of Biotechnology, College of Science and Biotechnology, Temple University, Philadelphia, PA, United States of America; University of Palermo, ITALY

## Abstract

Isolation of hepatocytes from cirrhotic human livers and subsequent primary culture are important new tools for laboratory research and cell-based therapeutics in the study of hepatocellular carcinoma (HCC). Using such techniques, we have previously identified different subpopulations of human hepatocytes and among them one is showing a progressive transformation of hepatocytes in HCC-like cells. We have hypothesized that increasing the distance from the neoplastic lesion might affect hepatocyte function and transformation capacity. However, limited information is available in comparing the growth and proliferation of human hepatocytes obtained from different areas of the same cirrhotic liver in relation to their distance from the HCC lesion. In this study, hepatocytes from 10 patients with cirrhosis and HCC undergoing surgical resections from specimens obtained at a proximal (CP) and distal (CD) distance from the HCC lesion were isolated and placed in primary culture. CP hepatocytes (CP-Hep) were isolated between 1 to 3 cm (leaving at least 1cm margin to avoid cancer cells and/or satellite lesions), while CD hepatocytes (CD-Hep) were isolated from more than 5 cm or from the contralateral-lobe. A statistical model was built to analyze the proliferation rates of these cells and we evaluated expression of HCC markers (Glypican-3 (GPC3), αSmooth Muscle Actin (α-SMA) and PCNA). We observed a significant difference in proliferation and in-vitro growth showing that CP-Hep had a proliferation pattern and rate significantly different than CD-Hep. Based on these data, this model can provide information to predict growth of human hepatocytes in primary culture in relation to their pre-cancerous state with significant differences in the HCC markers expression. This model provides an important innovative tool for in-vitro analysis of HCC.

## Introduction

Primary cultures of human hepatocytes (PHH) are a great resource for biomedical research and therapeutics purposes. [1–4] It is well accepted that a good portion of immortalized hepatocytes cell lines lack many typical aspects of primary cell function and consequently are questionable when used to evaluate mechanistic or therapeutic approaches. [5] The liver plays a crucial role in drug metabolism and for this hepatocytes are used to study the metabolic fate of drugs, drug-drug interactions and toxicity. [6–9] Different protocols have been developed to isolate hepatocytes from the liver and few studies showed that hepatocytes differ when isolated from healthy and diseased tissue [4, 5]. However, the isolation of hepatocytes from diseased liver is difficult and the overall results are variable in quality and viability, in particular in long term culture. [10]

To date, mostly immortalized cell lines have been used for the studies on HCC. Limited data exists on the use of hepatocytes isolated from the patients with cirrhosis and HCC and their maintenance in primary culture.

We have recently developed a new model for the study of HCC in-vitro and we demonstrated the existence of various human hepatocyte populations in the same liver which over time will transform into the cells with different morphologic and cancerous characteristics. [11] In this study, hepatocytes from patients with cirrhosis and HCC who had undergone surgical resections, were obtained from specimens at proximal (CP) and distal (CD) distances from the main HCC lesion and then were isolated and placed in primary culture. We showed that CP-Hepatocytes over a period of 16 weeks transformed into cells expressing high levels of markers for HCC (Glypican-3 (GPC3) and α Smooth Muscle Actin (α-SMA) and cell proliferation nuclear antigen (PCNA). They also developed invasiveness characteristics and gained the ability to aggregate in spheres that are well known characteristics of cancer cells with stem properties [11–12].

The purpose of this study was to analyze the relation between the specific HCC protein expressions in the CP, CD hepatocytes with changes in their cells growth rates over time. We hypothesized that the GPC3 expression will have a statistically significant correlation by the cultured cirrhotic cells over time.

This model provides significant hope that this method of study can offer new insights on the mechanisms associated with the transformation of hepatocytes in HCC compared to the study in-vitro of less dynamic immortalized cell lines. However, we believe that an accurate analysis of the proliferation characteristics of cells isolated from different locations of the cirrhotic liver and compared to their HCC like characteristics can provide information to predict growth of human hepatocytes in primary culture in relation to their pre-cancerous state providing an important innovative tool for in-vitro analysis of HCC.

## Materials and Methods

### Patients and sample collection

Research with Human Subjects and the Institutional Review Board (IRB) at University of Texas Medical Branch (UTMB) approved patients’ enrollment, consent procedures. Written consents were taken and recoded in electronic medical records in accordance with UTMB institutional policies. Samples were obtained from 10 patients with Hepatitis C which led to liver cirrhosis and solitary hepatic lesion. There were 9 man and 1 female among the patients with their age ranging from 49 to 69 years with the mean of 59.6. Tumor size were ranging from 1 to 7 cm with the mean of 2.76 cm. Histologically all tumors were well differentiated. According to TNM staging, seven were at T1, two at T2, and one at T3. No regional lymph node involvement (N0) and no metastasis (M0) was observed **(Table 1)**. (Control samples were taken from 5 patients (3 male and 2 female with mean age of 55.2) undergoing gallbladder removal surgery with no liver disease from pathological perspective. Fresh tissue samples were obtained at the time of surgery, immediately placed in cold (4°C) sterile saline solution and transported to the cell isolation laboratory. Histopathological evaluation of the tissue samples was performed using standard hematoxylin and eosin (H&E) staining. Sections were examined by a pathologist to confirm diagnosis of HCC and to rule out neoplastic contamination in cirrhotic liver samples utilized as cirrhotic proximal (CP) and cirrhotic distal (CD) for this study.

**Table 1:** Demographic–Pathologic characteristics of HCC patients (N = 10).

PATIENT#	Age/Sex	Tumor Size (cm)	Tumor Focality	Histologic Type of HCC	Grade	TNM staging	Tumor Extension	Cirrhosis	HCV/HBV
**1**	58/M	2.5	Solitary	Well differentiated	G1	T_1_N_x_M_0_	Confined to liver	Cirrhosis/ Steatosis	+/-
**2**	49/M	2	Solitary	Well differentiated	G2	T_2_N_x_M_0_	Confined to liver	Cirrhosis	+/-
**3**	68/M	2.1	Solitary	Well differentiated	G2	T_3_N_x_M_0_	Involved liver capsule	Cirrhosis	+/-
**4**	69/M	1	Solitary	Well differentiated	G1	T_1_N_x_M_0_	Confined to liver	Cirrhosis/ Severe Fibrosis	+/-
**5**	60/M	1	Solitary	Well differentiated	G2	T_1_N_x_M_0_	Confined to liver	Cirrhosis/ Severe Fibrosis	+/-
**6**	53/M	5.5	Solitary	Well differentiated	G3	T_2_N_0_M_N/A_	Confined to liver	Cirrhosis/ Steatosis	+/-
**7**	56/M	1	Solitary	Well differentiated	G2	T_1_N_x_M_N/A_	Confined to liver	Cirrhosis	+/-
**8**	63/M	3.5	Solitary	Well differentiated	G1	T_1_N_x_M_0_	Confined to liver	Cirrhosis	+/-
**9**	65/F	7	Solitary	Well differentiated	G1	T_1_N_x_M_N/A_	Confined to liver	Cirrhosis	+/-
**10**	55/M	2	Solitary	Well differentiated	G1	T_1_N_0_M_N/A_	Confined to liver	Cirrhosis	+/-

### Immunohistochemistry analysis

Specimens were fixed in 10% neutral formalin and embedded in paraffin. Tissue sections were cut at 3–5 μm and mounted on positively charged slides. Sections were treated with antigen retrieval to facilitate antibody binding to antigen and incubated in the Black and Decker Vegetable Steamer for 20 minutes in Target Retrieval Solution (Dako Corporation, Carpinteria, CA. Cat. #S1699) preheated to 99°C. When removed and cooled down, the slides were then rinsed three times with distilled water and placed into a container of Tris Buffered Saline with Tween 20 (Signet Pathology Systems, Inc., Dedham, MA. Cat. #2380). Both avidin and biotin (blocking kit; Vector Laboratories, Burlingame, CA. Cat. #SP2001) were diluted in Antibody Diluent (Dako) at a ratio of 1 ml avidin or biotin to 5 ml diluent. Diluted avidin was applied to sections and incubated for 7 minutes. The primary antibody was diluted to specific concentrations in the biotin solution and applied for the specific amount of time recommended by the company. Sections were then incubated in LSAB2, universal secondary antibody (Dako) for 15 minutes, followed by Chromagen liquid DAB (Dako) application for 5 minutes. Slides were taken off the Autostainer and rinsed in distilled water, manually counterstained with Harris Hematoxylin (Fisher Scientific,) for 1 minute, rinsed in distilled water followed by 0.25% ammonia water and rinsed in distilled water again. Following dehydration through graded series of alcohols, they were cleared in four changes of xylene and cover slipped with cover glass. Heppar1 was analyzed by immunohistochemistry. The antibodies and concentrations used were Heppar1 (1:50; Dako Clone OCH1E5 #M7158) and α-SMA (Abcam Mouse mAb 1A4 ab7817 1:200). For imunofuorescent tissue sections antibodies used are: Cytokeratin 18 (CK18, 1:100 [RGE53] ab9217 Abcam), Albumin (ALB, 1:250 (F-10) sc-271605, Santa Cruz), Arginase 1 (ARG1, 1:50 anti-liver Arginase [4E6] ab117989 Abcam), β-Catenin (1:250, [E247] ab 32572 Abcam), E-Cadherin (1:100, [M168] ab76055 Abcam) and c-Met (1:300, D1C2 #8189 Cell Signaling Technology).

### Immunofluorescence staining

Cells were fixed with 3.7% formaldehyde (Sigma-Aldrich) for 10 minutes at room temperature (r.t.) and permeabilized with 0.1% Triton X-100 (Sigma-Aldrich) in PBS for 5 minutes. Cells were then rinsed and covered with PBS blocking buffer (1% BSA in PBS) for 30 minutes at 37°C to minimize non-specific adsorption of the antibodies to the coverslips. After washing with PBS, cells were incubated with the primary antibodies (anti-GPC3 and anti-α-SMA, Abcam; diluted in PBS + 1% BSA + 0.05% NaN_3_) at 4°C, overnight. Preparations were washed three time with PBS and incubated for 1 hour at room temperature with secondary antibodies, either Alexa Fluor 488 (Abcam #150113) or Alexa Fluor 596 (Abcam #150080) diluted 1/1000 in 1% BSA + 0.05% NaN_3_. Nuclei were counterstained with 2.5 μg/ml Hoechst 33342 (Life Technologies NucBlue® Live ReadyProbes® Reagent; Grand Island, NY 14072 USA #37605), for 15–20 minutes. Following three washes with PBS, cells were examined on Olympus BX51 optic microscope equipped with fluorescence and suitable filters for Alexa Fluor 488, Alexa Fluor 596 and DAPI detection; images were captured and photographed using a computer-imaging system (PictureFrame^TM^). Primary antibodies used for cell immunofluorescence staining included: Heppar1 (1:100, DAKO M7158), CD68 (1:500, Santa Cruz USA, sc-393951), CD31 (1:500, Santa Cruz USA, sc-376764) and α-SMA (1:100, [1A4] ab 7817 Abcam).

### Isolation and in-vitro culture of primary CP, CD cells

Tissue specimens obtained were washed in PBS and processed within 2 hours from surgical resection. Samples were washed with physiologic solution, minced with fine sterile scissors and scalpel into fragments of approximately 1 mm^3^. Cells were immediately isolated from cirrhotic tissue proximal (: 1 < CP <3cm from the tumor resection margin) and distal (CD: >5cm from the tumor resection margin or contra-lateral lobe) to the HCC. The cell isolation procedure was performed as previously shown [11]. Briefly, the 1 mm^3^ fragments of tissue were incubated for 3 hours with Fetal Bovine Serum (FBS) HyClone (Fisher Scientific). FBS was then replaced by complete RPMI 1640 medium with 10% FBS, 1% of antibiotics (Corning-Cellgro) and amino acids (Sigma-Aldrich; MEM Non-essential amino acid solution (100×) #M7145) and incubated for 24 hours. Every 48 hours cells were then washed with 2 ml of RPMI 1640 complete medium. After 8 weeks a monolayer of primary cells around the explants was observed. Cells were detached using trypsin/EDTA 1X (Corning-Cellgro, USA), re-plated and maintained in culture at 37°C and 5% CO_2_.

### Soft Agar Colony Formation Assay

To exclude the presence of neoplastic cells in CP and CD tissue we tested cells isolated at 3 weeks with a soft agar colony formation assay. Cell were re-suspended in DMEM (Cell Biolabs CBA-140-T) supplemented with 6% FBS containing 0.4% agar. They were then seeded in three duplicate wells at a density of 5x10^4^ cells per well in a 12-well plate containing a bottom layer of DMEM supplemented with 10% FBS and 0.6% agar. Cell-agar suspension was overlaid with media containing 10% FBS and cultured for 7 days. After 7 days, the soft agar layer was solubilized, and spheres were collected and reseeded in RPMI 1640. At day 7 the size and number of spheres were calculated using Software ImageJ. The pictures of the spheres at day 7 were collected using a Nikon Eclipse TS100 optic microscope.

### CP and CD hepatocytes growth and data collection

Hepatocytes isolated from CP and CD tissue (CP-Hep and CD-Hep) were incubated from the time of resection up to 16 weeks. At week 8, 2.5x10^5^ cells of either CP-Hep or CD-Hep were collected. Cells were then detached at 2 weeks intervals (from week 10 to 16) with 1 X Trypsin EDTA and counted. The number of cells obtained for each time point was employed to develop a growth model for CP-Hep and CD-Hep, as reported in the statistical methods and results.

### Protein extraction and western blot analysis

Western blotting was performed on whole cell lysates to detect expression of GPC3 (Abcam Mouse mAb 9C2 ab129381 1:2000), α-SMA (Abcam Mouse mAb 1A4 ab7817 1:300) and PCNA (PC10 Mouse mAb #2586 1:2000). Cells were cultured and harvested before confluence. 1×10^7^ cells were lysed using a modified RIPA buffer [150 mM NaCl, 25 mMTris (pH 7.4), 1 mM EDTA, 1 mM EGTA, 2 mM Na3VO4, 10 mM NaF, 1% NP40, 10% glycerol, aprotinin (10 mg/ml) and leupeptin (10 mg/ml)]. Cell lysate was mantained for 15 minutes on ice and than extracts were centrifuged at 14,000 x g in a cold microcentrifuge for 10 minutes. Supernatant was collected and quantified by spectofotometry using BCA protein assay (Pierce, Rockford, IL). Equal amounts of proteins were separated by SDS-PAGE and transferred to nitrocellulose membrane (Li-Cor #926–31090), which was blocked using 5% non-fat dry milk in Tris-Buffered saline with Tween 20 (Blocking Buffer Li-Cor Biosciences; Lincon, USA # 927–40040). The membrane was incubated overnight at 4°C with the primary antibodies listed above. After incubation, the membrane was washed 3 times with PBST and then rinsed and incubated for 1h at r.t. in appropriate anti-mouse or anti-rabbit IRDye 680–800 secondary antibodies (Li-Cor Biosciences). The membrane was rinsed, developed with Odyssey Imaging Systems Li-Cor and specific protein bands were detected with Image Studio Software (Version 4.0.21 Li-Cor).GAPDH served as loading controls. Primary antibodies used for western blotting analysis: N-Cadherin (1:500 (H-2) sc-393933 Santa Cruz), E-Cadherin (1:1000, [M168] ab76055 Abcam), CK18 (5μg/μl [RGE53] ab9217 Abcam), Vimentin (1:2500, [EPR3776] ab92547 Abcam), β-Catenin (1:5000, [E247] ab 32572 Abcam), ALB (1:500, (F-10) sc-271605, Santa Cruz), ARG1 (1:1000, anti-liver Arginase [4E6] ab117989 Abcam), Id-1 (1:5000, [EPR7098] ab134163 Abcam), Glypican3 (1:1000, [9C2] ab129381 Abcam), α-SMA (1:150, [1A4] ab 7817 Abcam), PCNA (1:1000, (D3H8P) #13110 Cell Signaling Technology) and GAPDH (1:1000, (D16H11) #5174 Cell Signaling Technology).

### Statistical methods

The number of CP-Hep and CD-Hep of these ten patients were compared with the control group by t-tests. The differences in the number of CP- and CD-Hep over time have been compared using a newly developed growth model. Let *N*(*t*) be the number of counted cells at time *t*. As previously described, cells have been counted at time 1 (isolation of cells), at time 2 (10 weeks after), at time 3 (12 weeks after), at time 4 (14 weeks after) and at time 5 (16 weeks after) both in the two areas. The two available samples are not independent. Measurements are referred to cells obtained from two different areas of the same liver. Therefore, the number of cells between them have been compared by paired t-test. Normality of differences has been previously verified by comparing the empirical cumulative distribution function of the samples with the expected normal distribution (Anderson-Darling test). All the represented results were obtained from experiments repeated in triplicate for each sample obtained from each patient. Samples from 15 different patients were used in each experiment.

### Modeling cells growth

For each subject, cell growth, both in the proximal and distal area, is not inherently linear. It can be made to be linear after a transformation. In particular, an exponential model has been considered:

$$N\left(t\right)=\alpha e^{\gamma t}$$ (1)

where *t* is the observation time and *N(t)* represents the number of cells at that time. Taking the natural logarithm of both sides of the equation, the following holds:

lnN(t)=lnα+γt (2)

This equation has the form of a linear regression model that, after adding an error term *ε*, becomes

lnN(t)=lnα+γt+ε (3)

By considering y* = *lnN*(*t*) and α* = *lnα*, Eq (3) becomes

$$y^{*}=\gamma t+\alpha^{*}+\varepsilon$$ (4)

Such a model is referred to as a *log-level regression model*, where γ and α* can be determined with the available data. In order to determine if data follow this model, they have been previously log-transformed. As the linear regression model is significant for the log-transformed data, the cells growth model can be considered exponential. Additionally, an indication of the instantaneous rate of change in the number of cells can be addressed by the first derivative, $\frac{dN(t)}{dt}$, where *N*(*t*) is represented by Eq (1). It holds:

$$N^{\prime }\left(t\right)=\alpha \gamma e^{\gamma t}=e^{\alpha^{*}}\gamma e^{\gamma t}$$ (5)

### Growth Rate

Number of cells have been counted at a fixed step of two weeks. GR is defined as measure of the rate at which they increase in a given time period as a fraction of the initial population. Therefore, given a time period, *t*_*i*_ − *t*_*i*−1_ and indicating with *N*(*t*_*i*_) and *N*(*t*_*i*−1_) respectively the number of cells at time *t*_*i*_ and at time *t*_*i*−1_, the following can be calculated:

$$GR=\frac{N\left(t_{i}\right)-N\left(t_{i-1}\right)}{N\left(t_{i-1}\right)}$$ (6)

Or in percentage

$$GR=\frac{N\left(t_{i}\right)-N\left(t_{i-1}\right)}{N\left(t_{i-1}\right)}\times 100$$ (7)

If data are available at a fixed time, say T (the time window of interest), different from 1, Eq (6) needs to be divided for T. GRs have been calculated for the proximal and distal cells in the following time intervals, from week 8 to week 10, from week 10 to week 12, from week 12 to week 14 and, from week 14 to week 16. GRs related to the same time window have been compared by a paired t-test. An average growth rate (AGR) has also been computed and compared for the two populations referred to the total time window (from week 8 to week 16).

## Results

### Modeling cells growth results

The number of CP-Hep and CD-Hep have been compared by paired t-tests at four observation times (2, 3, 4 and 5 as described in statistical methods). For instance, time 1 has not been considered, because it represents the time in which the same number of cells has been assumed for all the subjects. Normality tests show that the distribution of differences between the number of CP-Hep and CD-Hep for each time is normal. Deviations from normality are not significant: time 2 (p-value = 0.333), time 3 (p-value = 0.916), time 4 (p-value = 0.553), time 5 (p-value = 0.901). Furthermore, in order to compare CD-Hep and CP-Hepatocytes with the control group, t-tests have been performed. All the differences are significant with a common p<0.0001 as shown in **Table 2**. The latter also shows the mean number of cells at the different time points for CP-Hep (CP-NC), CD-Hep (CD-NC) and for the control group (NL-Hep), the mean differences between CP-Hep and NL-Hep, CD-Hep and NL-Hep and between CD-Hep and CP-Hep. P-values are also reported with the 95% confidence intervals for the previous differences.

**Table 2:** Comparisons among number of cells for the following groups: CD-Hep, CP-Hep, NL-Hep. Number of patients: 10, number of subject in the control group: 5.

Time Points	CD-Hep	CP-Hep	NL-Hep	p-value
2	397770	420910	325000	
3	705635	747190	599400	
4	2161394	2167170	1223400	
5	4758900	6691460	2328200	
	**Difference (CD-Hep_CP-Hep)**	**95% CI for Difference**		
2	-23140	(-35030;-11250)		0.002
3	-41555	(-49853;-33257)		<0.0001
4	-5776	(-22184;10632)		0.446
5	-1932560	(-2076120;-1789000)		<0.0001
	**Difference (CD-Hep_NL-Hep)**	**95% CI for Difference**		
2	72770	(51270; 94270)		<0.0001
3	106235	(94498; 117972)		<0.0001
4	937994	(876645; 999343)		<0.0001
5	2430700	(2355783; 2505617)		<0.0001
	**Difference (CP-Hep_NL-Hep)**	**95% CI for Difference**		
2	95910	(74906; 116914)		<0.0001
3	147790	(138172; 157408)		<0.0001
4	943770	(883586; 1003954)		<0.0001
5	4363260	(4227639; 4498881)		<0.0001

These results are consistent with previous studies [11] and with the time of clinical observations of HCC recurrences after liver resection occurring approximately 3 months after the procedure [13]. Data shown in **Table 2**, both CD-Hep and CP-Hep, have been log-transformed (**Table 3**). For these transformed data the linear regression model has been found significant. Therefore, the mean cells growth model can be considered exponential.

**Table 3:** Number of cells for CD-Hep, CP-Hep and their log-transformed expression.

Time points	CD-Hep	log(CD-Hep)	CP-Hep	log(CP-Hep)
**1**	250000	5.398	250000	5.398
**2**	397770	5.600	420910	5.624
**3**	705635	5.849	747190	5.873
**4**	2161394	6.335	2167170	6.336
**5**	4758900	6.678	6691460	6.826

**Fig 1:**
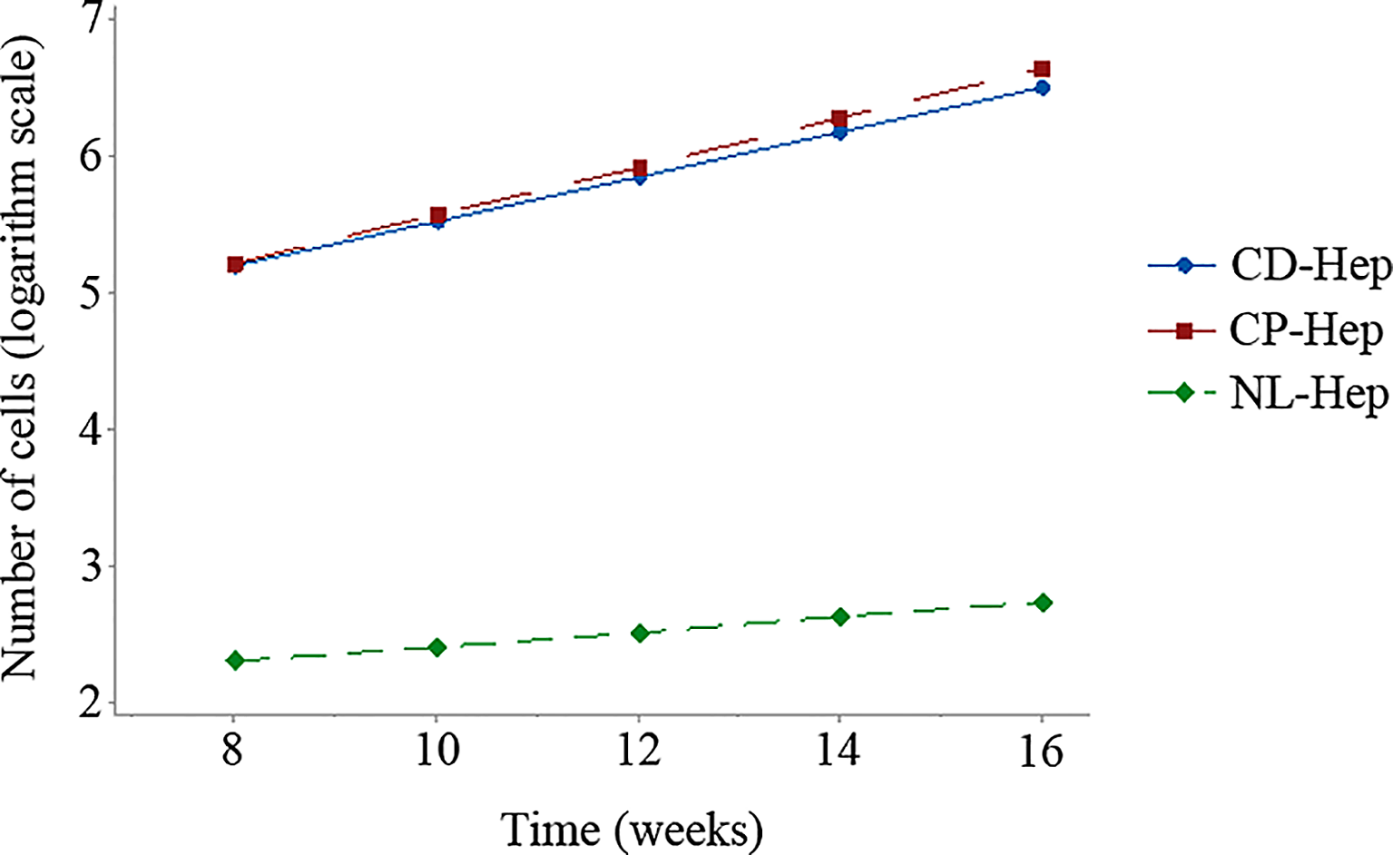
Number of CD-, CP- and NL-Hepatocytes over time in flash squares of 25^2 (logarithmic scale).

In particular, both for log(CD-Hep) and log(CP-Hep) the linear regression models are significant (p-value = 0.002). The coefficient of determination, R^2^, representing the proportion of the total variability explained by the regression model, is 97.52% and 95.82% respectively for the distal and the proximal models. Then, since the model with log transformed data is significantly linear, the growth of the number of cells is intrinsically exponential. As anticipated in section *Modeling cells growth*, the estimated parameters for the regression models α* and *γ* are respectively α* = 11.475 and *γ* = 0.7585 for the distal area and α* = 11.378 and *γ* = 0.8213 for the proximal area. In **Fig 1**, changes of the number of cells over time both for the distal and proximal areas and the control group are represented in a logarithmic scale. The cells growth trend for the control group is significantly reduced.

### Growth rate and average growth rate results

Growth rates have been computed according to the Eq (6) reported in the section Material and methods. Comparisons between CD-Hep and NL-Hep mean growth rates and between CP-Hep and NL-Hep mean growth rates were performed trough *t*-tests. Results, reported in **Table 4**, showed that differences for the first two groups are significant at time windows T1, T3 and T4. No difference is detected at time window T2. The same behavior is detected for CP-Hep and NL-Hep. Therefore, excluding time windows T2, mean cells growth rates are significantly lower for the control group. The different behavior at time window T2 is probably due to the increment and changes in hepatic metabolism of normal hepatocytes after the first month in culture. [14]

**Table 4:** Comparisons between the CD-Hep_NL-Hep and CP-Hep_NL-Hep mean growth rates.

Δt	Mean growth rate CD-Hep	Mean growth rate Control Group	*p-*value
**T1 (week 8 to 10)**	0.591	0.300	<0.0001
**T2 (week 10 to 12)**	0.777	0.848	0.211
**T3 (week 12 to 14)**	2.064	1.041	<0.0001
**T4 (week 14 to 16)**	1.202	0.906	0.003
	**Mean growth rate CP-Hep**		
**T1 (week 8 to 10)**	0.684	0.300	<0.0001
**T2 (week 10 to 12)**	0.776	0.848	0.197
**T3 (week 12 to 14)**	1.901	1.041	<0.0001
**T4 (week 14 to 16)**	2.088	0.906	<0.0001

**Fig 2:**
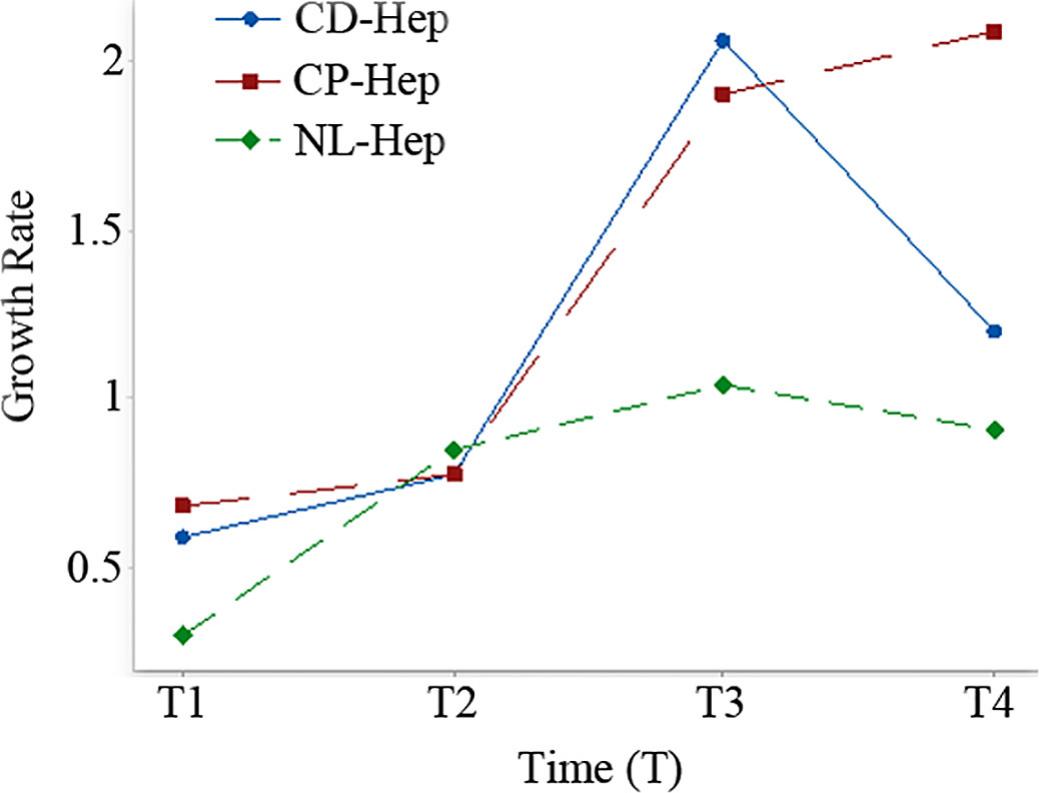
Mean growth rate in time windows of two weeks (T1, T2, T3 and T4) for CD-Hep, CP-Hep and NL-Hep.

In addition, differences between the proximal and the distal areas have been evaluated through paired t-tests. Results show that for the time windows T1 differences are significant (p-value = 0.002). The same for T3 and T4 (p-value<0.0001) while, for time window T2, differences are not significant (p = 0.990). The computed values of average growth rates are 1.158 for the distal area and 1.362 for the proximal one. In **Fig 2**the rate changes in those time domains are shown.

### In-vitro culture of hepatocytes and characterization

**Fig 3:**
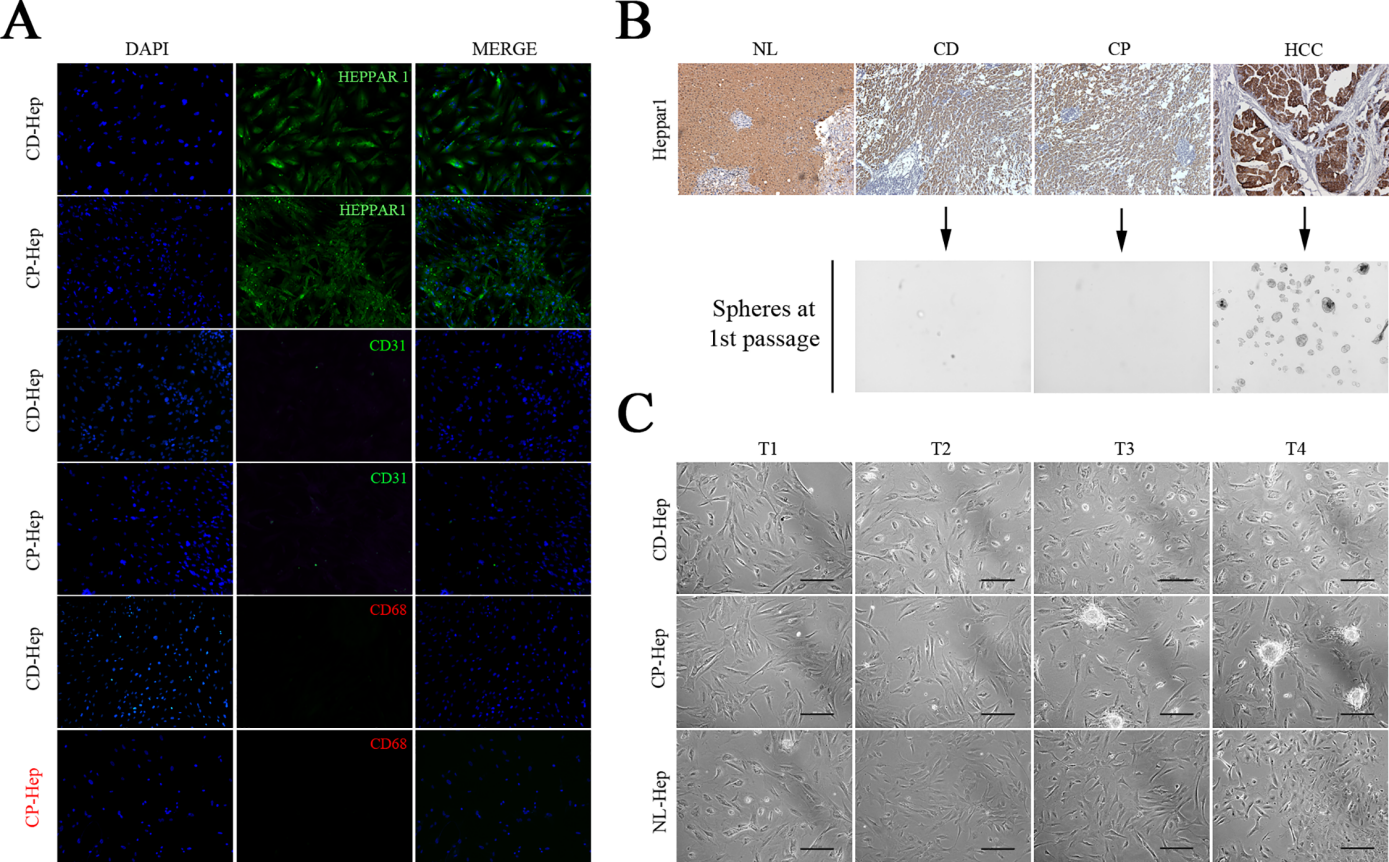
Morphological features of cultured cells obtained from CD, CP and NL tissue. A) Immunofluorescent staining in CD- and CP-Hep for Heppar1, CD31 (endothelial marker) and CD68 (macrophages marker) at 8 weeks. B) Representative immunohistochemical staining of Heppar1 in NL, CD, CP and HCC tissue utilized for cell isolation. Heppar1 showed overexpression in HCC compared to NL, CD and CP. C) Morphological features were observed at each time intervals (T1, T2, T3 and T4) and bright fields are captured (image magnification x100; black bar, 200μm).

**Fig 4:**
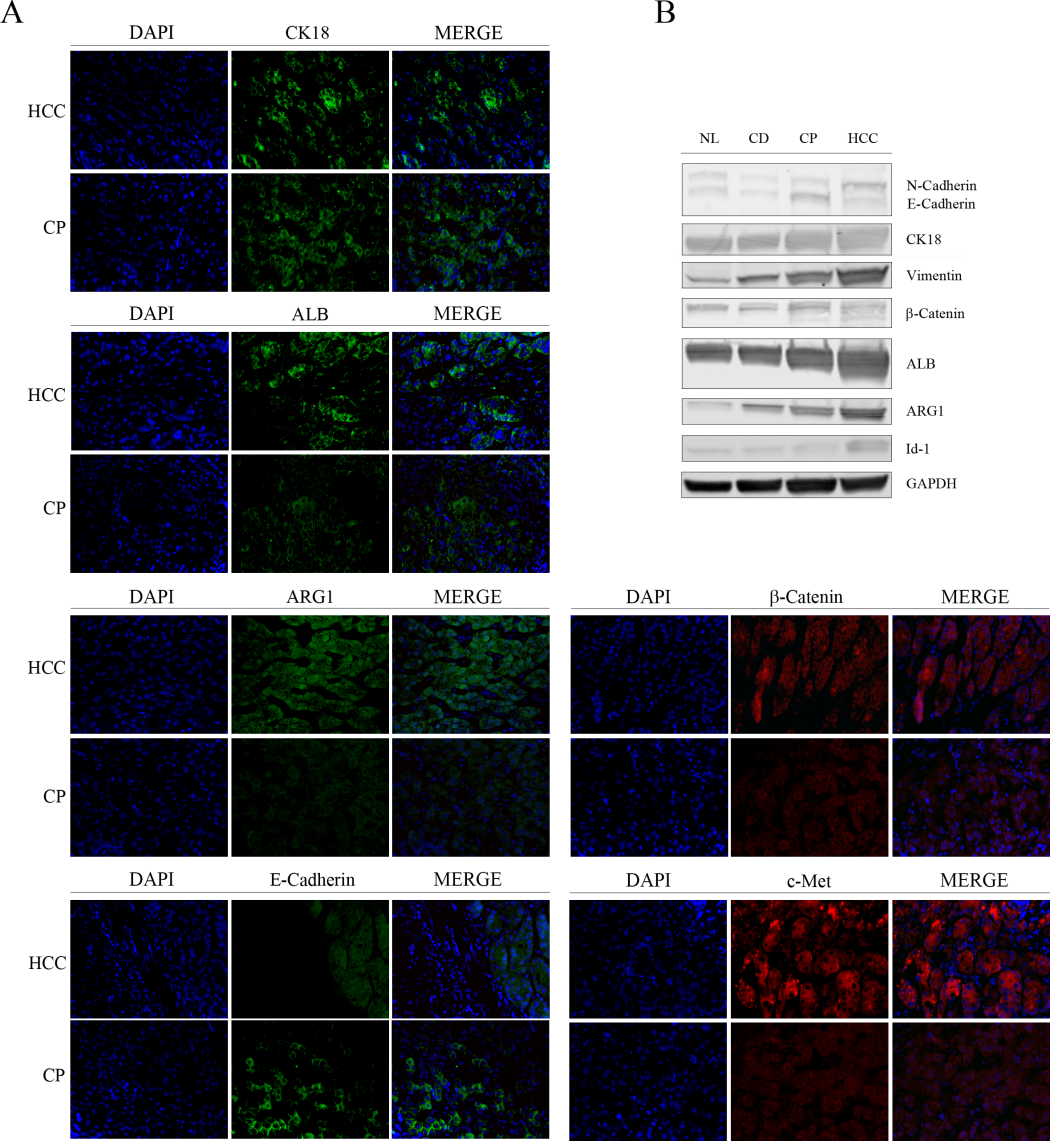
HCC markers expression. **A)** Immunofluorescent assay for CK18, ALB, ARG1, β-Catenin, E-Cadherin and c-Met. HCC and CP tissue were analyzed and we evidenced stronger signal in HCC than CP exception for E-Cadherin, highest in CP (image magnification x100). **B)** This difference in signal were confirmed by WB for N-Cadherin, E-Cadherin, CK18, Vimentin, β-Catenin, ALB, ARG1 and Id-1.

To evaluate the purity of isolated CD and CP cell cultures we detected positive cells for Heppar1 antigen with immunofluorescence, Heppar1 is expressed only from hepatocytes in liver tissue. [11] We observed a positivity of 93–96% showing an enrichment of hepatocytes in our cultures. We performed also Immunofluorescence for CD31 and CD68 to evidence contamination of endothelial and Kupffer cells, respectively. We did not observe macrophages and CD31 positive cells (**Fig 3A**). To avoid the presence on neoplastic cells (mainly from proximal tissue) from different area of cirrhotic livers we performed a immunohistochemistry for Heppar1 and histopathologic analysis confirmed absence of neoplastic cells, to improve our selection we performed a stringent transformation assay to detect neoplastic cells in our culture, we shown absence of spheres in culture from CD and CP districts indicating that at the moment of isolation hepatocytes are not transformed, while presence and high number of spheres were detected and observed in HCC cell culture as expected (**Fig 3B**), all cells (from CD and CP tissue) positive to transformation test were excluded from this study. In **Fig 3C** are showed bright fields of hepatocytes at each time intervals (T1, T2, T3 and T4). These results confirm that hepatocytes isolated from CD and CP tissue are not neoplastic, at the moment of isolation. To confirm the different phenotypes of hepatocytes isolated, we performed Immunohistochemistry and western blotting to evaluate the expression of HCC markers at 1st passage in-vitro culture (**Fig 4**) including Vimentin, CK18, ALB, ARG1 β-Catenin, c-Met and Id-1. Both immunohistochemistry and western blotting revealed that all markers were more intense in HCC cells compared to NL-, CD- and CP-Hep. We observed in HCC a decrement and different location of E-Cadherin compared to CP-Hep. In CP-Hep, E-Cadherin showed a canonical membrane location while it is cytoplasmic in HCC. We also observed an increment of N-Cadherin in HCC comparing with other samples. The contemporary over-expressions of CK18, Vimentin and N-Cadherin and down regulation of E-cadherin suggests an involvement of epithelia-mesenchymal transition in HCC carcinogenesis confirmed by morphological changes observed over time in-vitro culture (**Fig 3C**).

### Presence of Hepatic Stellate Cells in-vitro cell culture

**Fig 5:**
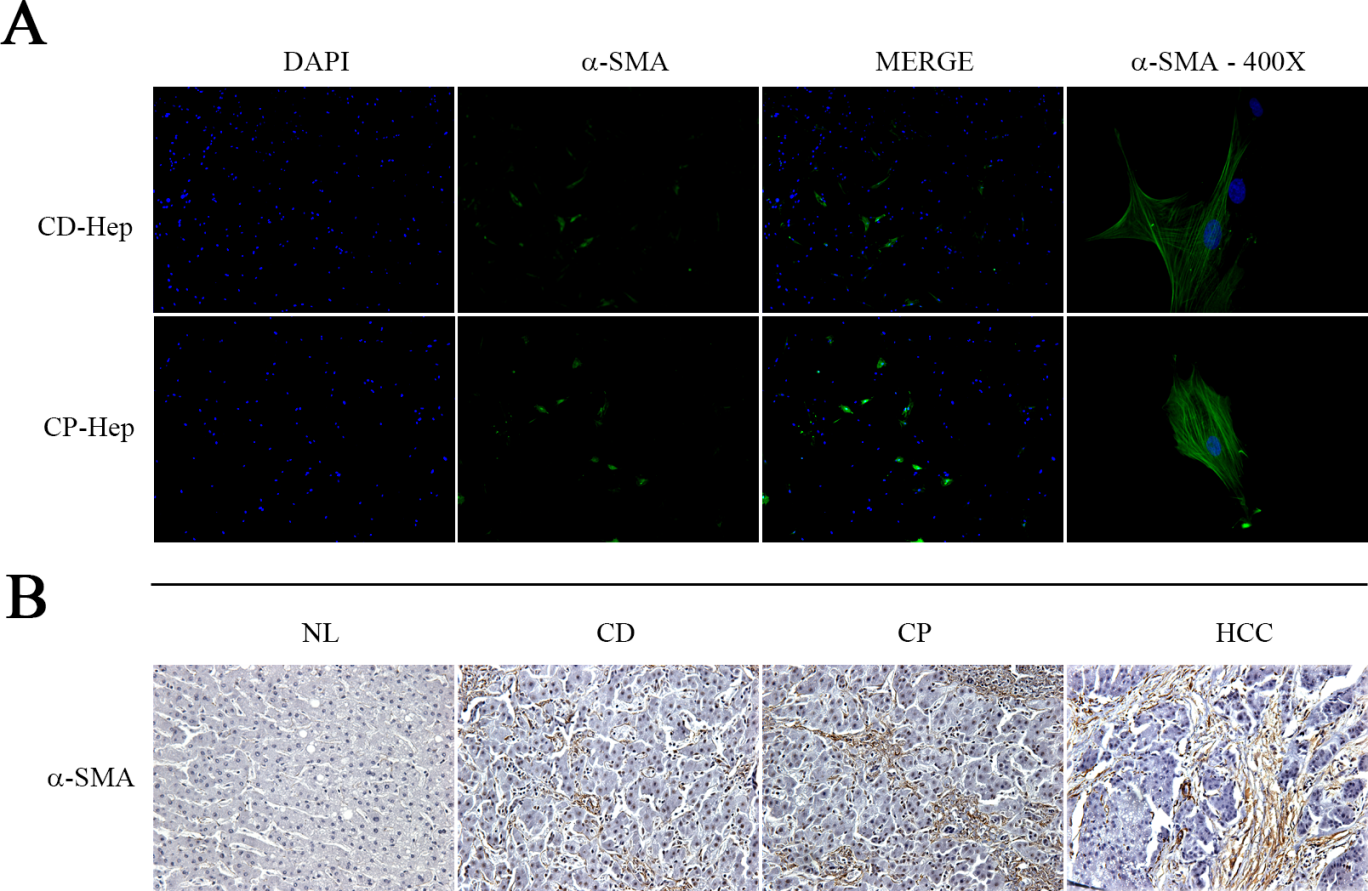
Hepatic Stellate Cells. **A)** Immunofluorescence with α-SMA detected positive cells in primary cultures in both cell populations (CD and CP, image magnification x40), α-SMA showed also its typical filamentous distribution in the cells, confirming their mesenchymal origin (image magnification x400). **B)** HSCs were observed also in tissue (NL, CD, CP and HCC) with immunohistochemistry analysis. Activated HSCs are absent in NL while as expected are activated and numerous in CD, CP and HCC tissue.

We observed in CP and CD cell culture the presence of hepatic stellate cells testing activation marker α-SMA with immunofluorescence (**Fig 5A**) and immunohistochemistry in NL, CD, CP and HCC tissue (**Fig 5B**). The immunohistochemistry showed the absence in NL and the positive signal in CD, CP and HCC as expected and showed previously [11]. Immunofluorescence confirmed the maintenance of activation state of these cells in cell culture isolated from CD and CP districts. Typical morphology and α-SMA distribution confirmed their presence (**Fig 5,** x400 image magnification).

### Expression of Glypican3, PCNA and α-SMA

**Fig 6:**
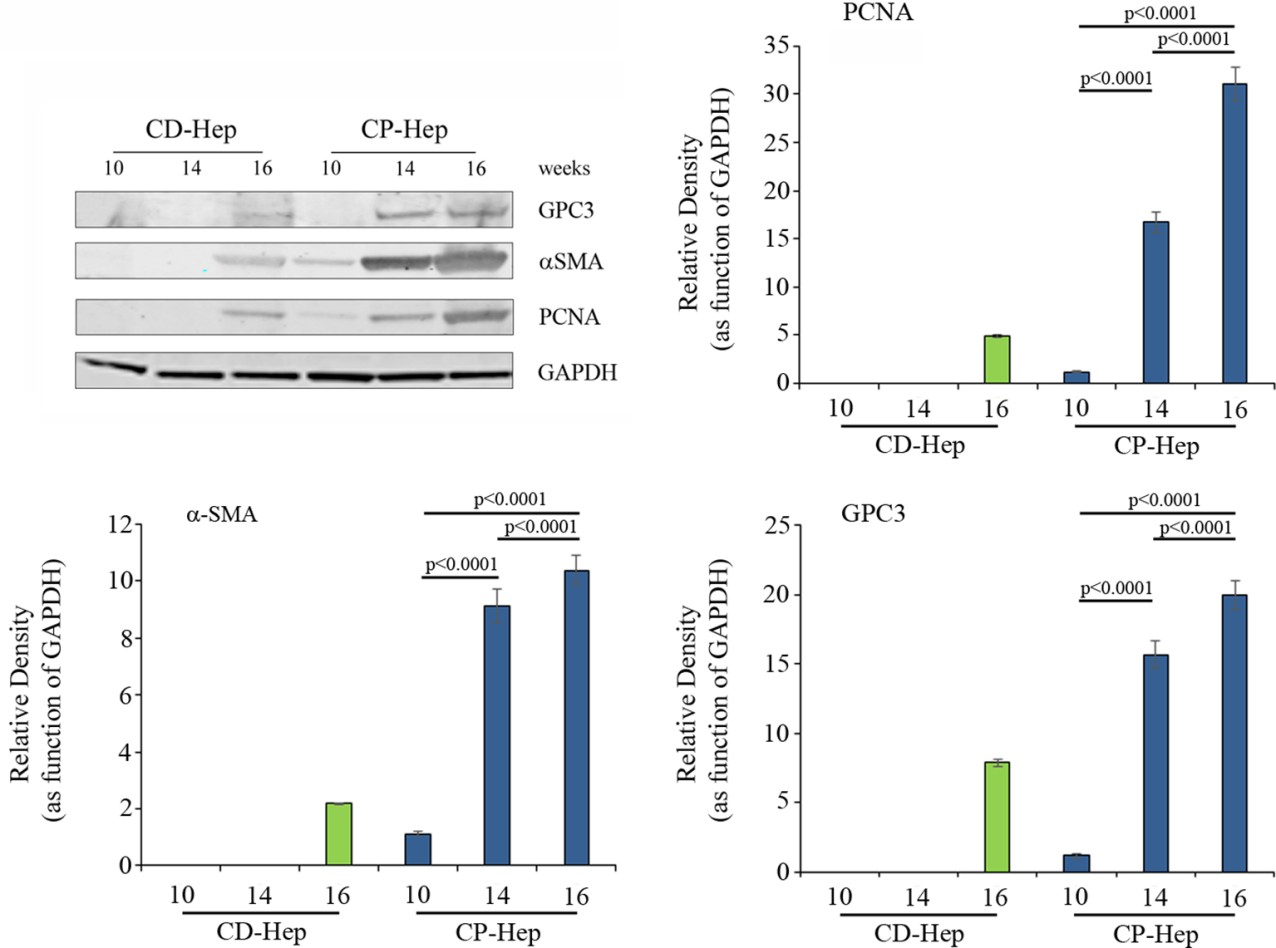
Expression of GPC3, α-SMA and PCNA. Expression of these markers were evaluated with western blotting. We showed that over time all of them are overexpressed, showing at each time point (10, 14 and 16 weeks) higher level in CP-Hep than CD-Hep. In CD-Hep we can appreciate expression of proteins only at 16 weeks. In CP-Hep for α-SMA we observed significant difference of expression (10 vs 14 p<0.0001; 10 vs 16 p<0.0001; 14 vs 16 p<0.0001) and also for GPC3 (10 vs 14 p<0.0001; 10 vs 16 p<0.0001; 14 vs 16 p<0.0001) and PCNA (10 vs 14 p<0.0001; 10 vs 16 p<0.0001; 14 vs 16 p<0.0001).

We observed that expression of Glypican3, PCNA and α-SMA is time dependent in-vitro culture (**Fig 6**). In particular all these proteins showed a similar behavior in each time point, even if with different protein levels. In particular, we observed that at each time point the level of these proteins is always significantly higher in CP- Hep than CD-Hep confirming that the hepatocytes have different phenotype influenced by the distance from HCC cell and its microenvironment and we showed that these differences are maintained in culture over time [11]. Glypican3 in CD-Hep is absent at week 10 and 14 and it is expressed at week 16 while CP-Hep express GPC3 at 10 weeks but the increment at 14 and 16 weeks is higher than in CD-Hep. The same behavior is observed for PCNA and α-SMA. For α-SMA we observed that its expression is HSCs-dependent and this suggest that close to the tumor there is an early intervention of HSCs even if we do not know what is exactly their function in diseased liver. This suggests a crucial role of HSCs for early manifestation of this cancer. All the difference in protein levels in GPC3, PCNA and α-SMA are significant at each time point suggesting a potential use of them as direct and indirect early diagnostic markers for HCC.

## Discussion

We previously showed that hepatocytes isolated from different areas of a single cirrhotic liver with HCC present different morpho-functional characteristics over time while cultured in-vitro. In particular, primary cultures of the cells obtained in closer proximity to HCC lesion seem to be already committed to become HCC and behave as precancerous hepatocytes [11].

In the present study we modeled the growth of CP-Hep and CD-Hep and analyzed their growth rates. From this analysis we observed that at week 16 proliferation rates of CP-Hep is significantly higher than CD-Hep while they were similarly lower up to week 14. Interestingly, this mimics the clinical progression of the disease. Following liver resection the average time of HCC recurrence is about 3 months and mostly in areas proximal to the resection margin that has been histologically reported to be negative. This suggests that pre-cancerous cells are probably present in the liver near the area of the primary HCC lesion at the time of resection and in about 3 month they develop into detectable as discrete HCC lesions of fully matured HCC.

In order to understand if the cells growth rates are accompanied by cellular cancerous transformation and therefore with the expression of proteins considered as HCC markers, levels of several proteins were calculated over time in culture. In CD-Hep we observed that protein expression appeared later and only on week 16 while in CP-Hep at week 10 for α-SMA, PCNA and GPC3. These timely fashioned differences in protein expression levels and cell growth rates can be translated into measure for early detection of tumor recurrence. For example, a measure amount of any of these markers: PCNA [15], GPC3 [16] and α-SMA [17] when compared before resection can lead clinicians to work up the patient more aggressively in order to find a recurrence.

## Abbreviations

HCC: hepatocellular carcinoma
GPC3: Glypican-3
FBS: fetal bovine serum
CP: cirrhotic proxima
CD: cirrhotic dista
NL: normal liver
PHH: primary human hepatocytes
CP-Hep: hepatocytes isolated from cirrhotic proximal tissue
CD-Hep: hepatocytes isolated from cirrhotic distal tissue
NL-Hep: hepatocytes isolated from normal liver
α-SMA: α-smooth muscle actin
BSA: bovine serum albumin
PCNA: proliferating cell nuclear antigen
AGR: average growth rate
ARG1: arginase1
ALB: albumin
CK18: cytokeratin18
